# Endothelial Iron Homeostasis Regulates Blood-Brain Barrier Integrity via the HIF2α—Ve-Cadherin Pathway

**DOI:** 10.3390/pharmaceutics13030311

**Published:** 2021-02-28

**Authors:** Daniel Rand, Orly Ravid, Dana Atrakchi, Hila Israelov, Yael Bresler, Chen Shemesh, Liora Omesi, Sigal Liraz-Zaltsman, Fabien Gosselet, Taber S. Maskrey, Michal Schnaider Beeri, Peter Wipf, Itzik Cooper

**Affiliations:** 1 The Joseph Sagol Neuroscience Center, Sheba Medical Center, Tel Hashomer 52621, Israel; Daniel.rand@sheba.health.gov.il (D.R.); Orly.Ravid@sheba.health.gov.il (O.R.); Dana.Atrakchi@sheba.health.gov.il (D.A.); hilaisraelov8@gmail.com (H.I.); yael.bresler@sheba.health.gov.il (Y.B.); Chen.Shemesh@sheba.health.gov.il (C.S.); Liora.Omesi@sheba.health.gov.il (L.O.); Sigal.LirazZaltsman@sheba.health.gov.il (S.L.-Z.); michal.beeri@mssm.edu (M.S.B.); 2 Sackler Faculty of Medicine, Tel-Aviv University, Tel Aviv 69978, Israel; 3 Department of Pharmacology, The Institute for Drug Research, The Hebrew University of Jerusalem, Jerusalem 97905, Israel; 4 Department of Sports Therapy, Institute for Health and Medical Professions, Ono Academic College, Kiryat Ono 55000, Israel; 5 Blood-Brain Barrier Laboratory (LBHE), Artois University, UR 2465, F-62300 Lens, France; fabien.gosselet@univ-artois.fr; 6 Department of Chemistry and Department of Bioengineering, University of Pittsburgh, Pittsburgh, PA 15260, USA; taber.maskrey@pitt.edu (T.S.M.); pwipf@pitt.edu (P.W.); 7 Department of Psychiatry, The Icahn School of Medicine at Mount Sinai, New York, NY 10029, USA; 8 School of Psychology, Interdisciplinary Center (IDC), Herzliya 4610101, Israel; 9 The Nehemia Rubin Excellence in Biomedical Research—The TELEM Program, Sheba Medical Center, Tel-Hashomer 5262000, Israel

**Keywords:** blood-brain barrier, iron, DFO, HIF2A, Ve-cadherin

## Abstract

The objective of this study was to investigate the molecular response to damage at the blood-brain barrier (BBB) and to elucidate critical pathways that might lead to effective treatment in central nervous system (CNS) pathologies in which the BBB is compromised. We have used a human, stem-cell derived in-vitro BBB injury model to gain a better understanding of the mechanisms controlling BBB integrity. Chemical injury induced by exposure to an organophosphate resulted in rapid lipid peroxidation, initiating a ferroptosis-like process. Additionally, mitochondrial ROS formation (MRF) and increase in mitochondrial membrane permeability were induced, leading to apoptotic cell death. Yet, these processes did not directly result in damage to barrier functionality, since blocking them did not reverse the increased permeability. We found that the iron chelator, Desferal© significantly decreased MRF and apoptosis subsequent to barrier insult, while also rescuing barrier integrity by inhibiting the labile iron pool increase, inducing HIF2α expression and preventing the degradation of Ve-cadherin specifically on the endothelial cell surface. Moreover, the novel nitroxide JP4-039 significantly rescued both injury-induced endothelium cell toxicity and barrier functionality. Elucidating a regulatory pathway that maintains BBB integrity illuminates a potential therapeutic approach to protect the BBB degradation that is evident in many neurological diseases.

## 1. Introduction

The blood–brain barrier (BBB) is composed of the capillaries of the central nervous system (CNS) which tightly regulate the movement of molecules, ions, and cells between blood and brain [1,2]. The BBB further protects the CNS by preventing the entry of neurotoxic plasma components and pathogens [3]. It is formed by a monolayer of tightly-sealed endothelial cells that make up the walls of the capillaries, and together with the closely associated pericytes and astrocytic end-feet processes control the restricted flow of compounds in and out of the brain, both through their paracellular junctions and by a limited array of transcellular vesicular and specialized transporters routes [4,5,6]. BBB breakdown and dysfunction leads to leakage of harmful blood components into the CNS, cellular infiltration, and aberrant transport and clearance of molecules [3,6,7]. Alzheimer’s disease (AD), amyotrophic lateral sclerosis (ALS) and Parkinson disease (PD) are all associated with defective BBB function [8,9,10,11,12]. In the aging brain, a region-specific increase of total iron content is observed, most likely triggered by inflammation, increased BBB permeability, redistribution of iron within the brain, and changes in iron homeostasis [13,14,15]. Changes in regional iron distribution have been demonstrated consistently in neurodegenerative diseases [16,17]. Additionally, many neurodegenerative diseases, such as AD and PD, are primarily characterized by a deposition of insoluble protein aggregates of which iron is an essential component [18,19,20,21]. Iron is seen as a potential biomarker for neurodegenerative diseases since increased iron levels appear early on in disease progression [22,23]. This phenomenon has raised the question of whether this iron increase is involved in disease initiation or is an early symptom of disease progression [24]. BBB breakdown has also been reported as an early physiological occurrence in these diseases and in ageing [25,26,27]. Yet, even though these closely associated events have been extensively researched, iron’s effect on BBB integrity and the molecular pathways that govern these effects still remain unclear, since the main emphasis in this field has been to understand iron transport through the BBB to the brain [28]. Another hallmark of these neurodegenerative disorders is an excessive amount of reactive oxygen species (ROS), which is viewed as one of the potential common etiologies in these diseases [29,30,31]. Imbalance between ROS production and antioxidant defenses results in excessive accumulation of ROS and leads to oxidative stress [32,33]. Oxidative stress results in cell membrane damage from lipid peroxidation, changes in protein structure and function due to protein degradation, and structural damage to DNA [34]. The brain is especially susceptible to oxidative stress due to its high oxygen demand, constituting 20% of body oxygen consumption [35]. Additionally, redox-active metals such as iron exist abundantly in the brain and are actively involved in the formation and propagation of ROS. Lastly, high levels of polyunsaturated fatty acids are present in brain cell membranes and form substrates for lipid peroxidation [36]. All these components seem to be interconnected, yet how they influence each other is still unclear. High iron levels, BBB permeability and oxidative stress are all biomarkers for neurodegenerative diseases [37,38,39] but thus far it is still uncertain whether these are isolated events or an interlinked physiological cascade. To understand the relationship and kinetics of these processes and their role in BBB breakdown we established a BBB-injury model based on the organophosphate paraoxon (PX) and a stem-cell derived in-vitro human BBB system [40,41,42,43,44]. Exposure to organophosphates induces common effects that are predominant in neurodegenerative diseases, such as; direct damage to BBB integrity [45,46] and ROS production among varied cell types, including endothelial cells [47,48,49]. Our model also exhibited many of these common BBB cellular abnormalities including increased permeability, ROS formation, inflammation, and cell death [43,44]. Since oxidative stress has been reported to be directly involved in BBB breakdown [34,50] we wanted to elucidate the specific ROS pathway in the endothelium responsible for this damage. To our surprise, we observed that blocking various ROS associated pathways such as lipid oxidation and mitochondrial superoxide formation had no significant effect on BBB integrity, although it rescued cells from cytotoxicity and apoptosis. Conversely, the FDA approved iron chelator Desferal (DFO) rescued the brain endothelial cells (BEC) from both toxicity and functional damage. We found that an increase in the BEC labile iron pool (LIP) was responsible for the BBB breakdown while DFO abrogated this effect by inhibiting the increase in LIP, inducing the expression of Hypoxia induced factor 2α (HIF2α) and reversing the loss of Ve-cadherin expression. HIF2α seems to be have an essential role in this process, since blocking it with a specific inhibitor abrogated the rescue effect of DFO on Ve-cadherin specifically at the cell surface but not on its overall expression. Additionally, the non-toxic nitroxide JP4-039 inhibited both BEC cell toxicity and rescued BBB integrity. These results have shed light on potential molecular targets and pathways to attenuate BBB breakdown, potentially contributing to therapeutic solutions in treatments of CNS pathologies where the loss of BBB integrity is an essential component.

## 2. Materials and Methods

### 2.1. Reagents

Mouse anti-VE-cadherin antibody (sc-9989) diluted 1:50 was obtained from Santa Cruz Biotechnology (Dallas, TX, USA). Cy and Alexa Fluor-conjugated secondary antibodies were acquired from Jackson Immunoresearch (Philadelphia, PA, USA) and Molecular Probes (Eugene, OR, USA), respectively, and used for immunocytochemistry. HIF2α inhibitor [51] (Axon 2034) was obtained from Axon Medchem (Hanzeplein, The Netherlands). Z-VAD-FMK was obtained from Adooq-bioscience (187389-52-2, Irvine, CA, USA) and Santa Cruz Biotechnology (sc-3067, Dallas, TX, USA). Marimastat from Santa Cruz Biotechnology (sc-202223, Dallas, TX, USA), MG-132 from Promega (G9951, Madison, WI, USA). Cytotoxgreen was obtained from Essen BioScience (Ann Arbor, MI, USA) and CellROXgreen from Molecular Probes (Eugene, OR, USA). PX-ethyl was purchased from Sigma (St. Louis, MO, USA). According to the Sigma safety data sheet, safety measures of eye shields, face shields, full-face respirator, and gloves should be taken. PX was used according to our previously reported protocol [43,44] All other reagents applied in this study were used in accordance to the known literature and the supplier’s guidelines.

### 2.2. JP4-039

JP4-039 is a drug-like small molecule that passes through Veber and Lipinski filters with a molecular weight (MW) of 424.6, a logP of 2.9, and a topological polar surface area (TPSA) of 70.7. Physicochemical properties were calculated with Instant JChem 21.2.0 (ChemAxon, Cambridge, MA, USA). The alkene peptide isostere has 5 hydrogen bond acceptors (HBA) and 2 hydrogen bond donors (HBD), as well as 9 rotatable bonds. Interestingly, a central nervous system (CNS) multiparameter optimization (MPO) generates a score of 4.6 for JP4-039 [52], which places this candidate in the upper cohort for drug-like properties, suggesting a desirable alignment of ADME attributes, blood−brain barrier crossing, and lower-risk safety profile. JP4-039’s aqueous solubility was determined to be 580 µM, and in a PAMPA permeability protocol, it was found to have a logPe (log of the effective permeability) of −4.2, suggesting high permeability [53]. JP4-039 was synthesized and analyzed as previously published [54].

### 2.3. Media

Brain-like endothelial cell (BLEC) and pericytes were grown in ECM medium (Sciencell, Carlsbad, CA, USA), which was composed as follows: 5% fetal calf serum (Gibco, Gaithersburg, MD, USA), ECGS supplements and 50 mg/mL gentamicin (Biological industries, Beit-Haemek, Israel).

### 2.4. BBB In-Vitro Model

To investigate the cellular response to chemical injury, a human BBB model, which is well characterized for studying BBB injury, inflammation, and barrier functionality was utilized [41,43]. The human BBB model was generated using human cord blood-derived from hematopoietic stem cells; CD34+ cells were isolated from umbilical cord blood. The parents of the infants signed a consent form. All protocols were done with the authorization of the French Ministry of Higher Education and Research (CODECOH Number DC2011-1321). CD34+ cells were differentiated into BLEC by a six days co-culture with bovine brain pericytes, as previously reported [41,43,44]. BBB injury was generated by exposure to the organophosphate PX, as previously characterized [43].

### 2.5. Permeability Assay

The protocol for fluorescence based permeability assay was used, as previously reported [43,44]. Fluorescein detection was carried out on an Infinite 200 PRO (Tecan, Männedorf, Switzerland) plate reader using the excitation/emission wavelength (nm) settings: 485/538. The Pe coefficient was obtained from the slope of the calculated clearance curve, as described in reference [55]. A typical Pe value for the control was Pe = 0.35 × 10^−3^ cm/min. 

### 2.6. TEER Assay

Impedance spectrum measurements were performed using a multi-well impedance spectrometer (cellZscope, Nano analytical, Berlin, Germany). Filter inserts, containing confluent monolayers of BLEC on the luminal side and pericytes on the abluminal side were placed in the cellZscope and impedance was measured at an hour interval for 12 h prior to treatment and 24 h post treatment, while being incubated at 37 °C and 5% CO_2_. TEER values in the current study reached an average of 35 ± 5 Ω·cm^2^ before treatments were applied.

### 2.7. Cell Death by LDH Release

The toxicity of PX was investigated on monolayers of BLEC seeded on 96 well plates or on inserts at the luminal side in co-culture experiments using the commercially available Cytotoxicity Detection Kit (Promega, Madison, WI, USA) according to manufacturer protocol. Absorption was measured at 490 nm by an Elisa plate reader (Tecan, Männedorf, Switzerland).

### 2.8. Cell Death by Cytotoxgreen Staining

Monolayers of BLEC were treated with PX in 96 well plates and monitored simultaneously for cytotoxic response kinetics with cytotox green stain (250 nM) for 24 h by live imaging using an IncuCyte imaging system (Essen BioScience, Ann Arbor, MI, USA). Images were captured every hour and cells with a positive fluorescence signal were counted by the IncuCyte^®^ integrated analysis software.

### 2.9. Oxidative Stress Analysis

Cellular oxidative stress was detected with the use of the cell-permeable fluorogenic probe CellROX (5 mM; Molecular Probes). Monolayers of BLEC were treated in 96 well plates and monitored with CellROX stain for 24 h by live imaging using an IncuCyte imaging system (Essen BioScience). The intensity of CellROX fluorescence was calculated and analyzed by the IncuCyte integrated analysis software to quantify ROS levels.

### 2.10. Apoptosis Analysis

Apoptosis was measured with the use of the apoptosis cell-permeable non-toxic fluorogenic Caspase-3/7 Reagent (Essen BioScience). Monolayers of BLEC were treated in 96-well plates and monitored with Caspase-3/7 Reagent for 24 h by live imaging using an IncuCyte imaging system (Essen BioScience). Images were captured every hour and cells with a positive fluorescence signal were counted by the IncuCyte integrated analysis software.

### 2.11. Mitochondrial ROS Formation Analysis

Superoxide formation in the mitochondria was measured with the use of the MitoSOX™ Red reagent (Molecular Probes) according to manufacturer protocol. Monolayers of BLEC were treated in 96-well plates and monitored with MitoSOX™ for 24 h by live imaging using an IncuCyte imaging system (Essen BioScience). Images were captured every hour and the fluorescence signal intensity was calculated and analyzed by the IncuCyte integrated analysis software to quantify mitochondrial ROS formation levels.

### 2.12. Mitochondrial Membrane Depolarization Analysis

Mitochondrial Membrane Depolarization was measured with the use of the membrane-permeant JC-1 dye (Molecular Probes) according to manufacturer protocol. Monolayers of BLEC were treated in 96-well plates and monitored with the JC-1 dye for 24 h by live imaging using an IncuCyte imaging system (Essen BioScience). Images were captured every hour and the increase in the green/red intensity fluorescence signal ratio was calculated and analyzed by the IncuCyte integrated analysis software to quantify mitochondrial membrane depolarization.

### 2.13. Lipid Peroxidation Analysis

Lipid peroxidation was measured with the use of C11-BODIPY^581/591^ (Molecular Probes) based upon the protocol previously reported [56]. Monolayers of BLEC were treated in 96 well plates and monitored with C11-BODIPY^581/591^ for 24 h by live imaging using an IncuCyte imaging system (Essen BioScience). Images were captured every hour and the increase in the green/red intensity fluorescence signal ratio was calculated and analyzed by the IncuCyte integrated analysis software to quantify lipid peroxidation. Additionally, the lipid peroxidation assay kit (Abcam, Cambridge, MA, USA) was also used according to the manufacturer protocol.

### 2.14. Labile Iron Pool Measurements

Cellular labile iron pool levels were measured with the use of calcein acetoxymethyl ester (CA-AM, Sigma-Aldrich, St. Louis, MO, USA) based upon protocols previously reported [57,58,59]. Monolayers of BLEC were treated in 96-well plates and simultaneously incubated with CA-AM (1 μM). Following staining, cells were analyzed by live imaging using an IncuCyte imaging system (Essen BioScience). Images were captured every hour and the increase in the green intensity fluorescence signal was calculated and analyzed by the IncuCyte integrated analysis software to quantify relative labile iron pool concentration.

### 2.15. Western Blot Assay

BLEC were grown on gelatin-coated 6-well plates and treated for 24 h. The cells were then lysed with RIPA Buffer (Sigma, USA) and protease inhibitors (Roche, City, France). Cell extracts were separated by 10% SDS–PAGE followed by blotting on nitrocellulose (Whatmann Schleicher-Schuell, City, France) and analyzed with a mouse anti-Ve-cadherin (Santa Cruz Biotechnology), and normalized with a rabbit anti-actin (Santa Cruz Biotechnology) antibodies. Original western blotting was cropped to remove an experimental treatment that was not incorporated in the results.

### 2.16. Immunocytochemistry

BLEC were immunostained, as previously reported [43,44]. In short, cells were fixed with 4% PFA, then incubated overnight at 4 °C with anti-VE-cadherin and anti-HIF2α antibodies, and washed with PBS/0.1% Tween20 and incubated with appropriate secondary antibodies for 1 h at room temperature. Cells were counterstained with propidium iodide (PI) for 5 min. Images were taken with IncuCyte^®^ fluorescence microscope (Essen BioScience) with ×10 objective. Antibody intensity fluorescence signal was calculated and analyzed by the IncuCyte integrated analysis software and normalized by cell count using the PI signal.

### 2.17. Gene Expression—rtPCR

BLEC were grown on gelatin-coated 6-well plates and treated for 24 h. RNA isolation was performed using the RNA Purification kit (NucleoSpin^®^) according to manufacturer protocol. The cDNA was synthesized using the qScript™ cDNA Synthesis Kit (Quanta Bioscience™). To precisely quantify specific mRNA expression, RT-PCR Step One Plus system (#8024, Applied Biosystems, Waltham, MA, USA) was used. Primers for Ve-cadherin and GAPDH (housekeeping gene) were used as previously reported [43,44].

### 2.18. Flow Cytometry (FACS)

BLEC were grown on gelatin-coated 6-well plates and treated for 24 h. After treatment, BLEC were collected using Cell Dissociation Non-enzymatic Solution (Sigma), washed, and suspended in cold PBS. One million (1 × 10^6^) cells were incubated with Anti-Ve cadherin Alexa Fluor 488 conjugated or isotype control (mouse IgM, Invitrogen, Waltham, MA, USA) for 40 min on ice. After three washes with cold PBS, cells were examined in the CyFlow^®^ Cube 6 (Sysmex America, Lincolnshire, IL, USA) and data was analyzed using the FlowJo™ v10.7 software (BD biosciences, San Diego, CA, USA).

### 2.19. Data and Statistical Analysis

Results are expressed as the mean ± standard deviation of the mean, with at least three biological repeats unless mentioned otherwise. Comparison between three groups or more was analyzed using one-way analysis of variance (ANOVA) with Tukey’s multiple comparison test for post-hoc analyses. Live cell imaging data was analyzed with two-way ANOVA with Tukey’s multiple comparison test for post-hoc analyses. GraphPad Prism 8.0 software was used for all statistical analysis (GraphPad Software Inc., La Jolla, CA, USA). Differences were considered significant at *p* values < 0.05.

## 3. Results

### 3.1. Lipid Peroxidation Is Rapidly Induced in a BBB Injury Model

**Figure 1:**
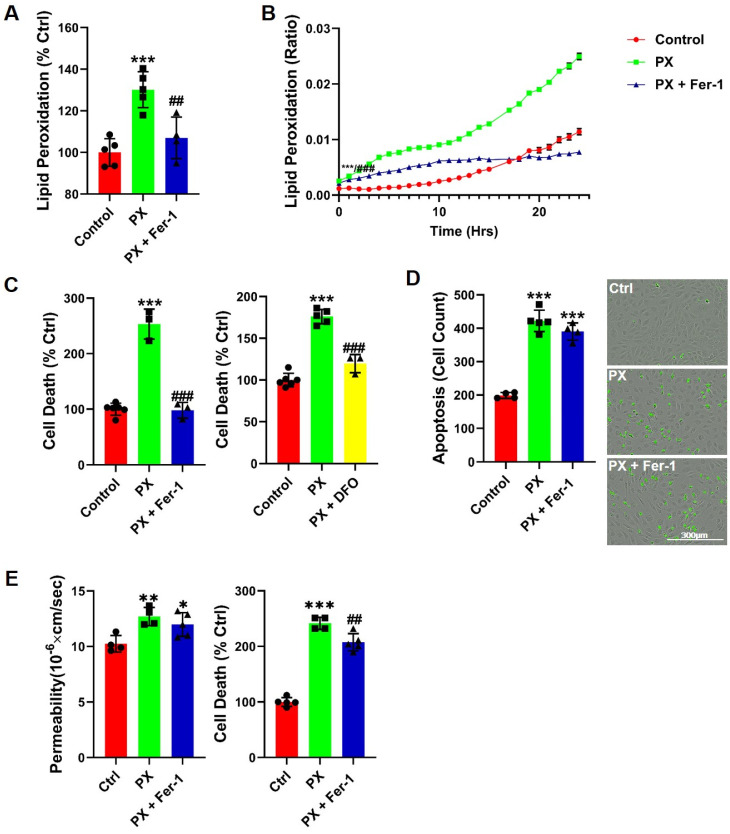
Inhibition of lipid peroxidation rescues cell viability but not barrier function. (**A**) Cellular lipid peroxidation 1-h subsequent to 400 µM PX treatment on a BLEC monolayer. Fer-1 was added at 50 µM. Ctrl are cells cultured with normal medium without the addition of any treatments. Mean ± s.d. of one representative experiment is shown, out of three independent experiments with *n* = 9–12, *** *p* < 0.001 vs. ctrl and ## *p* < 0.01 vs. PX; (**B**) Cellular lipid peroxidation was assessed by live cell imaging assay over a 24-h period on a BLEC monolayer. Mean ± s.d. of *n* = 4–6 for each condition from a single experiment, *** *p* < 0.001 vs. ctrl and ### *p* < 0.001 vs. PX marks the first time point with a significant change; (**C**) Cell death was assessed by the release of lactate dehydrogenase (LDH) 24-h post 400 µM PX treatment on a BLEC monolayer. Fer-1 was added at 50 µM and DFO was added at 50 µg/mL. Mean ± s.d. of one representative experiment is shown, out of three independent experiments with *n* = 10–18, *** *p* < 0.001 vs. ctrl and ### *p* < 0.001 vs. PX; (**D**) Apoptosis was measured with a caspase-3 activated fluorescent marker 24-h post PX treatment on a BLEC monolayer. Mean ± s.d. of one representative experiment is shown, out of three independent experiments with *n* = 9–12, *** *p* < 0.001 vs. ctrl; (**E**) Permeability of sodium fluorescein (NaF) was measured across the BBB in vitro model (from luminal to abluminal side) 24-h post PX treatment. PX was added at 900 µM and Fer-1 was added at 50 µM. Cell death was assessed by sampling medium from the luminal side with the LDH assay. Mean ± s.d. of one representative experiment is shown, out of three independent experiments with *n* = 9–15, * *p* < 0.05, ** *p* < 0.01 and *** *p* < 0.001 vs. ctrl and ## *p* < 0.01 vs. PX. Ctrl, control; PX, paraoxon; Fer-1, Ferrostatin; DFO, Desferal.

We have previously reported that neither antioxidants nor apoptosis inhibitors were able to rescue barrier functions in our BBB injury model [43,44], suggesting that the source of this damage is not a direct result of either process. We aimed to ascertain whether inhibiting a specific type of oxidative stress in the BLEC would attenuate the damage to BBB integrity. Since lipid compositions and their physiochemical properties greatly influence barrier functions [60] and intercellular lipids are a common target for oxidation by ROS [61,62], we measured lipid peroxidation at the initial insult event. A rapid and significant increase in lipid peroxidation was observed (Figure 1A). Additionally, we measured lipid peroxidation over a 24-h period using a live-cell analysis system and found that lipid peroxides accumulated over time (Figure 1B). The lipid peroxidation inhibitor Ferrostatin-1 (Fer-1) [63] abrogated this damage throughout the entire experiment (Figure 1A,B). Cell death was abrogated in the presence of Fer-1, indicating that lipid peroxidation contributed to the increased cell toxicity (Figure 1C). These results suggested that insult to BLEC might lead to ferroptosis for which Fer-1 is a classic and specific blocker [63]. The ferroptotic cell death entails two essential components: lipid peroxidation and accumulation of free iron ions [63,64]. We therefore treated cells with Desferal (DFO), an FDA-approved drug which acts as an iron chelator. Indeed, DFO significantly rescued cell toxicity (Figure 1C). To further confirm the ferroptosis process, the absence of apoptosis needed to be established [63,65]. The kinetics of apoptosis induction were measured with live cell imaging of caspase-3 activation for the duration of 24 h subsequent to endothelium exposure to PX. We found that PX induced apoptosis in BLEC, and that the addition of Fer-1 did not abrogate this effect (Figure 1D). Therefore, even though most of the classic ferroptosis signatures existed, it was inaccurate to classify this process as ferroptosis per se since ultimately, apoptosis occurred (Figure 1D). We then asked whether this ferroptosis-like process could account for the impaired functionality observed in the BBB model; Even though Fer-1 abrogated cell death (Figure 1C), it did not rescue barrier function from increased permeability (Figure 1E). Collectively, these results suggested that there were at least two processes that occurred as a result of PX damage to the brain endothelial cells. Initially, the cells rapidly responded to the PX challenge by lipid peroxidation; however, after 12 h, additional cell death pathways came into play, such as apoptosis. These seemed to be independent pathways, since BLEC underwent apoptosis even in the presence of Fer-1 (Figure 1D). Additionally, neither ferroptosis nor apoptosis seemed to be solely accountable for increased BBB permeability since two classic blockers of these programmed cell death processes (Fer-1 and the pan caspase inhibitor ZVAD) significantly attenuated cell death but failed to rescue the damage to barrier permeability (Figure 1E and [43]).

### 3.2. Kinetics of Mitochondrial ROS Formation

**Figure 2:**
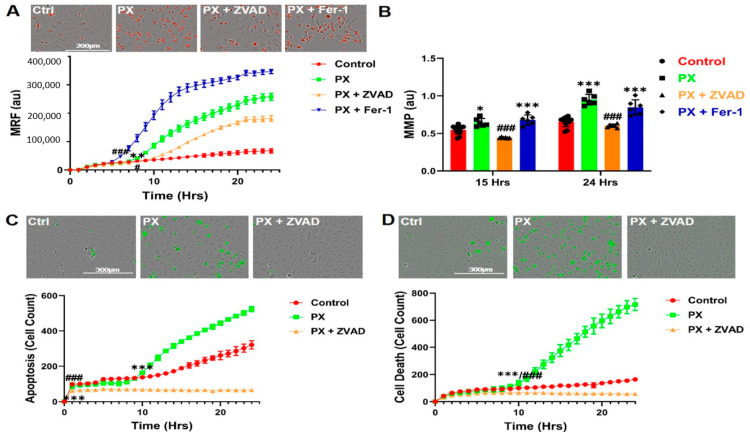
Apoptosis and mitochondrial stress are not a direct result of the early lipid peroxidation initiated by PX. (**A**) Live cell imaging of mitochondrial ROS formation was measured 24-h post 400 µM PX treatment on a BLEC monolayer. ZVAD and Fer-1 were added at 50 µM. Ctrl are cells cultured with normal medium without the addition of any treatments. Mean ± s.d. of one representative experiment is shown, out of three independent experiments with *n* = 12–18, ** *p* < 0.01 vs. ctrl and ### *p* < 0.001 vs. PX marks the first time point with a significant change; (**B**) Mitochondrial membrane permeability was examined by measuring its membrane depolarization with JC-1 staining over a 24-h period on a PX treated BLEC monolayer. Mean ± s.d. of one representative experiment is shown, out of three independent experiments with *n* = 12–18, * *p* < 0.05 and *** *p* < 0.001 vs. ctrl and ### *p* < 0.001 vs. PX; (**C**) Apoptosis was measured with a caspase-3 activated fluorescent marker by live cell imaging 24-h post PX treatment on a BLEC monolayer. ZVAD was added at 50 µM. Mean ± s.d. of one representative experiment is shown, out of three independent experiments with *n* = 9–12, *** *p* < 0.001 vs. ctrl and ### *p* < 0.001 vs. PX marks the first time point with a significant change; (**D**) Live cell imaging of cell death was examined by cytotoxicity fluorescent staining over a 24-h period on a PX treated BLEC monolayer. Mean ± s.d. of one representative experiment is shown, out of three independent experiments with *n* = 12–18, *** *p* < 0.001 vs. ctrl and ### *p* < 0.001 vs. PX marks the first time point with a significant change. Ctrl, control; PX, paraoxon; ZVAD, Z-VAD-FMK; Fer-1, Ferrostatin; MRF, mitochondrial ROS formation; MMP, mitochondrial membrane permeability; au, arbitrary units.

Since Fer-1 failed to inhibit PX-induced apoptosis (Figure 1D), we assumed that apoptosis was not a direct result of the initial lipid peroxidation. Since inhibition of several oxidative stress associated events in our model, such as lipid peroxidation (Figure 1B) and total cell ROS formation [43] did not rescue the BBB integrity, we next measured mitochondrial ROS production. Mitochondrial superoxide formation was significantly increased 9 h after PX treatment in a time dependent manner (Figure 2A). Mitochondrial stress initiates the chain of events leading to apoptosis [66], we therefore treated the cells with ZVAD, which significantly decreased PX-induced mitochondrial ROS formation, while Fer-1 exacerbated it (Figure 2A), suggesting that this organelle-specific ROS formation might be responsible for the chain of events leading to apoptosis. It has been reported that mitochondrial superoxide formation leads to a decrease in mitochondrial membrane potential, resulting in mitochondrial membrane permeation [67] which is the next step in the chain of events terminating in apoptosis. We therefore performed a live cell imaging assay to measure the permeation of the mitochondrial membrane in cells treated with PX. At 15 h post treatment, the permeation started to increase significantly in comparison to the control cells, increasing until 24 h. ZVAD abrogated this effect and Fer-1 had no significant effect at 24 h (Figure 2B). Additionally, ZVAD prevented the activation of caspase-3 and reduced the basal apoptosis process of untreated cells after 24 h (Figure 2C). Concordantly, ZVAD nullified cell cytotoxicity throughout the 24-h treatment (Figure 2D). Together, these results suggest two chain of events and their kinetics in this model of injured endothelium; one results in apoptosis while the other leads to a ferroptosis-like process. However, even though these experiments shed light on both chains of events and provided insights into their time frames, the source of functional damage in the brain endothelium is yet to be clarified.

### 3.3. The Iron Chelator DFO Rescues Both Cell Viability and Functionality

**Figure 3:**
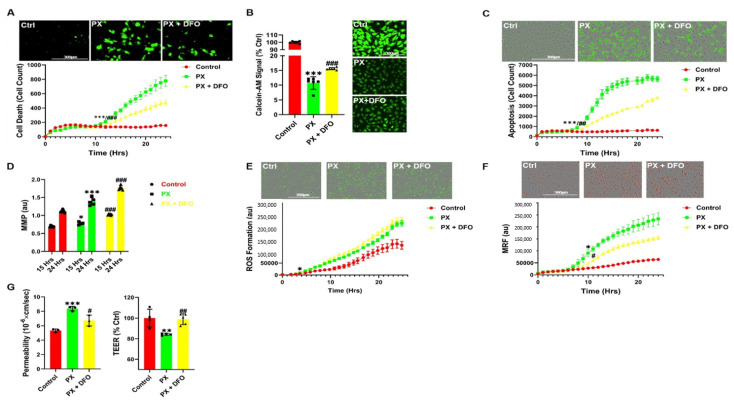
DFO diminishes cell toxicity and rescues barrier functions. (**A**) Live cell imaging of cell death was examined by cytotoxicity 24 h post 400 µM PX treatment on a BLEC monolayer. DFO was added at 50 µg/mL. Ctrl are cells cultured with normal medium without the addition of any treatments. Mean ± s.d. of one representative experiment is shown, out of five independent experiments with *n* = 20–30, *** *p* < 0.001 vs. ctrl and ## *p* < 0.01 vs. PX marks the first time point with a significant change; (**B**) Labile iron pool (LIP) was assessed by Calcein-AM on a PX treated BLEC monolayer 1-h post treatment. Its fluorescent signal negatively correlates with LIP. Mean ± s.d. of one representative experiment is shown, out of two independent experiments with *n* = 10–12, *** *p* < 0.001 vs. ctrl and ### *p* < 0.001 vs. PX; (**C**) Apoptosis was measured with a caspase-3 activated fluorescent marker by live cell imaging 24-h post PX treatment on a BLEC monolayer. Mean ± s.d. of one representative experiment is shown, out of three independent experiments with *n* = 12–18, *** *p* < 0.001 vs. ctrl and ## *p* < 0.01 vs. PX marks the first time point with a significant change; (**D**) Mitochondrial membrane permeability was evaluated with the use of live imaging by measuring the mitochondrial membrane depolarization with JC-1 staining on a PX treated BLEC monolayer. Mean ± s.d. of one representative experiment is shown, out of three independent experiments with *n* = 12–18, * *p* < 0.05 and *** *p* < 0.001 vs. ctrl and ### *p* < 0.001 vs. PX; (**E**) Live cell imaging of cellular ROS formation was measured with CellRox staining on a PX treated BLEC monolayer Mean ± s.d. of one representative experiment is shown, out of three independent experiments with *n* = 12–18, * *p* < 0.05 vs. ctrl marks the first time point with a significant change; (**F**) Live cell imaging of mitochondrial ROS formation was measured with the Mitosox marker on a PX treated BLEC monolayer. Mean ± s.d. of one representative experiment is shown, out of four independent experiments with n = 16-24, * *p* < 0. 05 vs. ctrl and # *p* < 0.05 vs. PX marks the first time point with a significant change; (**G**) Permeability of sodium fluorescein (NaF) and TEER were measured across the BBB in vitro model (from luminal to abluminal side) 24-h post PX treatment. PX was added at 900 µM and DFO added at 50 µg/mL. Mean ± s.d. of one representative experiment is shown, out of three independent experiments with *n* = 12–15, *** *p* < 0.001 and ** *p* < 0.01 vs. ctrl and # *p* < 0.05 and ## *p* < 0.01 vs. PX. Ctrl, control; PX, paraoxon; DFO, Desferal; MRF, mitochondrial ROS formation; MMP, mitochondrial membrane permeability; au, arbitrary units; TEER, Trans endothelial electrical resistance.

Since several ROS processes were evident in our model, but had no direct effect on BBB integrity, we wanted to elucidate the effect of DFO in our model since it rescued BLEC from PX-induced cell death (Figure 1B). Indeed, DFO rescued cytotoxicity throughout the 24-h window (Figure 3A). Since DFO is an iron chelator, we hypothesized that damage to BLEC deregulated the cellular iron homeostasis. Indeed, PX increased the labile iron pool within the cell, while DFO moderately but significantly rescued this effect (Figure 3B). Unlike Fer-1, DFO did not rescue the cells exclusively through the ferroptosis-like process since it also significantly decreased apoptosis (Figure 3C). Since apoptosis was inhibited, we next measured the effect of DFO on mitochondrial membrane permeation, a hallmark of the apoptosis pathway [68]. Conversely, DFO exacerbated the damage to the mitochondrial membrane (Figure 3D), suggesting that DFO did not affect apoptosis through the classic chain of events that are associated with the breakdown of the mitochondrial membrane. We next measured the cellular ROS formation since the Fenton reaction is a catalytic process in which iron is oxidized by hydrogen peroxide, a product of mitochondrial oxidative respiration, ultimately leading to the creation of a highly toxic hydroxyl free radical, which results in oxidative stress [69]. Surprisingly, DFO had no significant effect on cellular ROS formation (Figure 3E). It has been reported that the Fenton reaction initiates the production of hydroxyl radicals within the mitochondria [70], leading to oxidative stress. Indeed, DFO significantly decreased mitochondrial ROS formation (Figure 3F), suggesting that DFO rescues cell viability through inhibition of mitochondrial superoxide formation. Conversely, DFO significantly increased the permeation of the mitochondrial membrane rather than preventing it (Figure 3D) indicating that DFO does not rescue cell viability via the classic apoptosis pathway. Our next step was to test whether the ability of DFO to rescue cell viability in BLEC also extended to their functionality. DFO significantly rescued the barrier function as manifested by preventing the increase in permeability and the decrease in transendothelial electrical resistance (TEER) in the BBB model exposed to PX (Figure 3G). This confirms that DFO rescues both cell viability and barrier functionality. Collectively, these results highlight the therapeutic potential of addressing iron dyshomeostasis in the treatment of BBB damage since this approach has the dual effect of restoring loss of function as well as inhibiting cell toxicity in the endothelial cells of the BBB.

### 3.4. DFO Rescues BBB Integrity by Protecting Ve-Cadherin through Regulation of HIF2α

**Figure 4:**
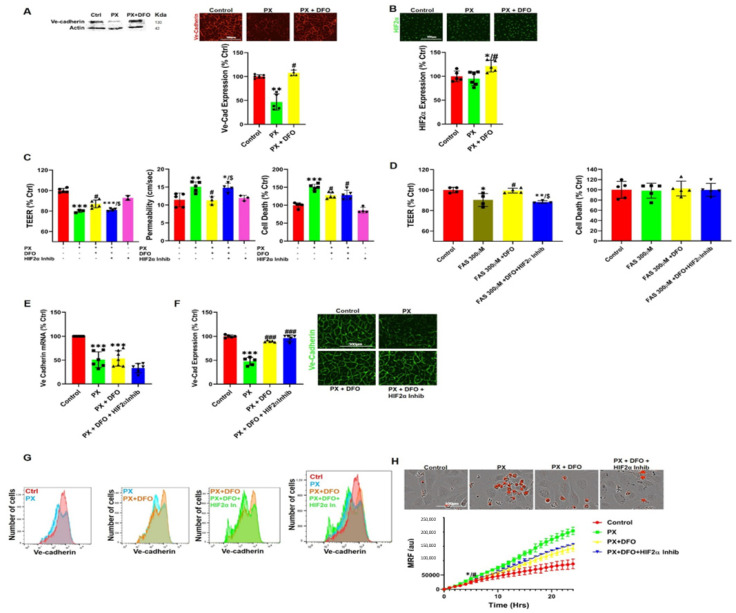
DFO rescues BBB Permeability via a HIF2α-Ve-cadherin pathway. (**A**) Ve-Cadherin expression was measured with western blotting and immunocytochemistry intensity quantification in a BLEC monolayer treated with 400 µM PX for 24 h. DFO was added at 50 µg/mL. Ctrl are cells cultured with normal medium without the addition of any treatments. Mean ± s.d. of one representative experiment is shown, out of four independent experiments with *n* = 16–24, ** *p* < 0.01 vs. ctrl and # *p* < 0.05 vs. PX. Scale bar = 200 µm; (**B**) Immunofluorescence staining and quantification of HIF2α in a BLEC monolayer treated with PX. Mean ± s.d. of one representative experiment is shown, out of four independent experiments with *n* = 16–24, * *p* < 0.05 vs. ctrl and # *p* < 0.05 vs. PX. Scale bar = 200 µm; (**C**) Permeability (10^6^ xcm/s) of sodium fluorescein (NaF) and TEER were measured across the BBB in vitro model (from luminal to abluminal side) 24-h post PX treatment. PX was added at 900 µM, DFO was added at 50 µg/mL and HIF2α inhibitor was added at 10 µM. Cell death was assessed by sampling medium from the luminal side with the LDH assay. Mean ± s.d. of one representative experiment is shown, out of three independent experiments with *n* = 9–15, *** *p* < 0.001, ** *p* < 0.01 and * *p* < 0.05 vs. ctrl and # *p* < 0.05 vs. PX and $ *p* < 0.05 vs. PX + DFO; (**D**) TEER was measured across the BBB in-vitro model 12-h post FAS treatment. FAS was added at 300 µM, DFO was added at 50 µg/mL, and HIF2α inhibitor was added at 10 µM. Cell death was assessed by sampling medium from the luminal side with the LDH assay. Mean ± s.d. of one representative experiment is shown, out of two independent experiments with *n* = 6–10, ** *p* < 0.01 and * *p* < 0.05 vs. ctrl and # *p* < 0.05 vs. FAS and $ *p* < 0.05 vs. FAS + DFO; (**E**) RT-PCR quantification of Ve-Cadherin mRNA in a BLEC monolayer treated with 400 µM PX for 24 h. DFO was added at 50 µg/mL and HIF2α inhibitor was added at 10 µM. Mean ± s.d. of *n* = 5–6 for each condition from six biological repeats and three technical repeats per each n, *** *p* < 0.001 vs. ctrl; (**F**) Immunofluorescence staining and quantification of Ve-Cadherin in a BLEC monolayer treated with PX for 24 h. Mean ± s.d. of one representative experiment is shown, out of four independent experiments with *n* = 16–24, *** *p* < 0.001 vs. ctrl and ### *p* < 0.001 vs. PXScale bar = 300 µm; (**G**) FACS histograms of live single cells from a BLEC monolayer treated with PX for 24 h. Cells were stained for Ve-Cadherin. Immunostaining was performed without fixation and permeabilization (surface staining), *n* = 3 for each condition from 3 independent experiments. Median decrease of 32% with PX treatment (*p* < 0.001), DFO rescue of 22% (*p* < 0.01) and HIF2α inhibitor attenuated DFO rescue by 34% (*p* < 0.01); (**H**) Live cell imaging of mitochondrial ROS formation was measured with Mitosox staining on a BLEC monolayer treated with PX for 24 h. Mean ± s.d. of one representative experiment is shown, out of three independent experiments with *n* = 12, * *p* < 0.05 vs. ctrl and # *p* < 0.05 vs. PX marks the first time point with a significant change. Scale bar = 100 µm. Ctrl, control; PX, paraoxon; DFO, Desferal; HIF2α, Hypoxia induced factor; FAS, ferrous ammonium sulfate; TEER, Trans endothelial electrical resistance; MRF, mitochondrial ROS formation; au, arbitrary units.

Next, we investigated the effect of DFO on adherens and tight junctions, which are responsible for the paracellular tightness of the BBB. Out of all the adherens and tight junctional proteins we evaluated; Ve-cadherin expression was the most affected by exposure to PX in-vivo and in-vitro [43,44]. Additionally, Ve-cadherin is known to be a master regulator of permeability in BBB endothelial cells [71]. We therefore measured Ve-cadherin expression in cells exposed to a combined treatment of PX and DFO. Using western blotting and immunocytochemistry, DFO was shown to abrogate the reduction in total Ve-cadherin protein expression (Figure 4A). To investigate a connection between the increased labile iron pool and Ve-cadherin down regulation, we measured the levels of the iron regulated transcription factor; hypoxia induced factor 2 alpha (HIF2α). This transcription factor has been implicated as a Ve-cadherin regulator [72,73]. While there was no significant effect on HIF2α expression in the injury model, addition of DFO significantly increased HIF2α expression levels in comparison to Control and PX-treated cells (Figure 4B). These results translated exclusively to the functional level since the HIF2α inhibitor abrogated the rescue effect of DFO on both permeability and TEER but not cell viability (Figure 4C). This strengthened the hypothesis that the labile iron pool is responsible for the damage to BBB integrity since this pattern replicated itself when PX was exchanged with ferrous ammonium sulfate (FAS) to directly increase the labile iron pool [74] while not inducing cell toxicity (Figure 4D). HIF2α is a transcription factor that specifically induces Ve-cadherin expression independently of hypoxia [73]. We therefore measured mRNA levels in BLEC exposed to PX. Surprisingly, even though HIF2α inhibitor had an adverse yet not significant effect on Ve-cadherin levels, DFO did not rescue mRNA decrease in response to insult (Figure 4E), suggesting that DFO rescued Ve-cadherin expression post transcriptionally. We then measured the effect of HIF2α on Ve-cadherin whole cell protein levels and found that even though DFO prevented the decrease in Ve-cadherin protein expression, HIF2α inhibitor did not abrogate this effect (Figure 4F). Conversely, Ve-cadherin cell surface expression reflected a clear pattern of DFO rescue and HIF2α inhibitor attenuation of this rescue effect (Figure 4G). To ascertain that this functional pathway is independent of the ROS associated apoptotic pathways mentioned earlier, we measured MRF. As expected, HIF2α inhibitor had no significant effect on DFO rescue (Figure 4H), strengthening the hypothesis that endothelium functionality is not regulated by ROS related processes in our model. Collectively, these results suggest that HIF2α regulates BBB integrity through maintaining Ve-cadherin on the cell surface. Damage to the BBB increases the cellular labile iron pool, resulting in a decrease in HIF2α-mediated Ve-cadherin cell surface expression, while DFO restores this expression, resulting in the functional rescue of the BBB endothelial cells.

### 3.5. A New Chemical Entity, JP4-039, Rescues Barrier Functions

**Figure 5:**
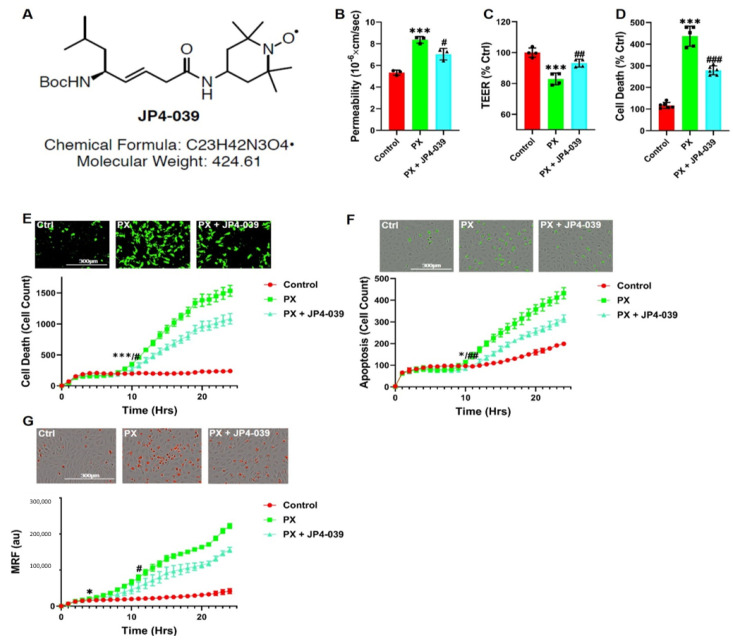
JP4-039 rescues PX-induced toxicity and permeability increase in the BBB injury model; (**A**) A chemical formula diagram of JP4-039; (**B**) Permeability of sodium fluorescein (NaF) was measured across the BBB in vitro model (from luminal to abluminal side) 24-h post PX treatment. PX was added at 900 µM and JP4-039 was added at 25 µM. Ctrl are cells cultured with normal medium without the addition of any treatments. Mean ± s.d. of one representative experiment is shown, out of four independent experiments with *n* = 12–20, *** *p* < 0.001 vs. ctrl and # *p* < 0.05, vs. PX; (**C**) Using the same model, TEER was also measured. Mean ± s.d. of one representative experiment is shown, out of four independent experiments with *n* = 12–20, *** *p* < 0.001 vs. ctrl and ## *p* < 0.01 vs. PX; (*D*) Cell death was also assessed in the same model by the release of lactate dehydrogenase (LDH).Mean ± s.d. of one representative experiment is shown, out of three independent experiments with *n* = 9–18, *** *p* < 0.001 vs. ctrl and ### *p* < 0.001 vs. PX; (**E**) Live cell imaging of cell death was examined by cytotoxicity fluorescent staining 24-h post 400 µM PX treatment on a BLEC monolayer. JP4-039 was added at 624 nM. Mean ± s.d. of one representative experiment is shown, out of four independent experiments with *n* = 16–24, *** *p* < 0.001 vs. ctrl and # *p* < 0.05 vs. PX marks the first time point with a significant change; (**F**) Apoptosis was measured with a caspase-3 activated fluorescent marker by live cell imaging 24-h post PX treatment on a BLEC monolayer. Mean ± s.d. of one representative experiment is shown, out of four independent experiments with *n* = 16–24, * *p* < 0.05 vs. ctrl and ## *p* < 0.01 vs. PX marks the first time point with a significant change; (**G**) Live cell imaging of mitochondrial ROS formation was measured with Mitosox staining on a BLEC monolayer treated with PX for 24 h. Mean ± s.d. of one representative experiment is shown, out of five independent experiments with n = 20–30, * *p* < 0. 05 vs. ctrl and # *p* < 0.05 vs. PX marks the first time point with a significant change. Ctrl, control; PX, paraoxon; TEER, Trans endothelial electrical resistance; MRF, mitochondrial ROS formation; au, arbitrary units.

Since the cellular iron homeostasis is a precise and delicately regulated process that is present throughout all cells in the body [75,76], we aimed at identifying another agent that does not elicit such a profound effect on the cellular iron levels. Similar to DFO, this compound should have the potential to mitigate the synergistic effects of increased cell death and decreased cell functionality induced in the damaged BBB. We found that the novel nitroxide, JP4-039 (Figure 5A), significantly rescued permeability in our injury model (Figure 5B) and inhibited TEER decrease (Figure 5C). JP4-039 also inhibited cell death as recorded using the LDH assay (Figure 5D). The cytotoxicity and apoptosis kinetics were measured in the presence of JP4-039. Both processes were initially inhibited at 8 to 10 h and up to 24 h post treatment (Figure 5E,F). We have previously shown that JP4-039 is an antioxidant and electron scavenger that targets the mitochondria [77]. Indeed, JP4-039 significantly inhibited MRF increase (Figure 5G) in PX treated cells. Collectively, these results suggested that the novel nitroxide, JP4-039, offers a therapeutic potential to treat BBB damage, since this compound has the dual effect of rescuing both the viability and integrity of the endothelial cells.

## 4. Discussion

**Figure 6:**
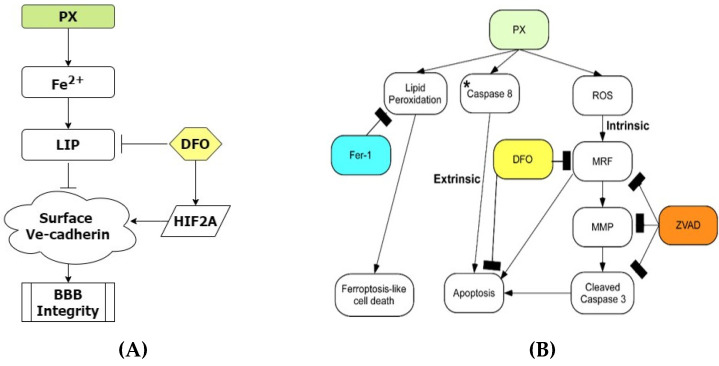
BBB integrity is regulated by the iron dependent HIF2α-Ve cadherin axis. (**A**) Flowchart elucidating the molecular pathways that regulate BBB integrity in our model. Upon insult the labile iron pool is significantly increased, resulting in damage to the BBB integrity. DFO rescues BBB functionality by inducing the expression of HIF2α that results in the restoration of Ve-cadherin on the cell surface; (**B**) Flowchart elucidating the molecular pathways initiated in our model. * We have recently reported that inhibiting caspase-8 decreased apoptosis in our model [44]. PX, paraoxon; ZVAD, Z-VAD-FMK; Fer-1, Ferrostatin; MRF, mitochondrial ROS formation; MMP, mitochondrial membrane permeability; au, arbitrary units.

In this study, we have used a human stem cell-derived BBB injury model to elucidate molecular mechanisms and their kinetics that are induced upon insult with the organophosphate PX. High correlation has been demonstrated in humans between cognitive decline, neurodegenerative diseases, and exposure to organophosphates [78,79,80,81], and several in vivo studies have shown that BBB integrity is compromised after exposure to organophosphates [82] and specifically to PX [46]. Most in-vivo experiments examining chemical toxins effects on BBB have been performed on rodents, especially experiments elucidating ROS and oxidative stress effects on the BBB [83,84,85]. Yet, rodents BBB differ from that of humans by their permeability to P-glycoprotein substrates [86] and expression levels of tight junctions, such as claudin-5 [87] and receptors, such as Transferrin Receptor1 [88,89]. Since our model is based on human cord blood derived hematopoietic stem cells that have been co-cultured with pericytes to differentiate into BBB endothelium, the conundrum of discrepancies between species has been addressed. Additionally, this in-vitro human BBB model correlated well with human pharmacokinetic parameters [41]. As we previously reported, both the apoptosis inhibitor ZVAD and the antioxidant TEMPOL abrogated PX-induced endothelial cell viability damage; yet, they had no significant effect on the reduced barrier functionality [43]. It has been widely reported that ROS affects BBB integrity in animals [84] and in-vitro [90,91] as well as under varying pathologies such as stroke, traumatic brain injury (TBI) and neurodegenerative diseases [92,93,94]. Thus, we decided to elucidate whether a more organelle-specific oxidative stress was responsible for BBB functional damage. Since membrane phospholipids are sensitive to ROS attacks [95] and it has been previously reported in other BBB models that lipid peroxidation damages BBB integrity [60], we measured lipid peroxidation and examined its effects on BBB integrity. A rapid increase in lipid peroxidation was evident upon insult accumulating over 24 h (Figure 1A,B). It has been previously reported that lipid peroxidation induces ferroptosis, a relatively new non-apoptotic, iron-dependent regulated form of cell death caused by the accumulation of lipid-based ROS [63]. Even though there have been several studies in neurons on this process [96,97,98], no study to date has been undertaken to elucidate its role in BBB endothelium. To ascertain whether this programmed cell death was activated in our model, we added both the classic ferroptosis and lipid peroxidation inhibitor; Ferrostatin-1 and the iron chelator DFO. Both compounds attenuated cell viability damage (Figure 1C). To validate ferroptosis, the absence of apoptosis needs to be confirmed. Even though caspase-3 activation was recorded in conjunction with significantly increased cell death 10 h post insult (Figure 2D), Fer-1 had no effect on apoptosis (Figure 1D). These findings negate the possibility of the classic ferroptosis being induced, since ultimately the cells underwent apoptosis. Recent reports have highlighted ferroptosis ability to sensitize the cell to compound-induced apoptosis, such as the tumor necrosis factor related apoptosis-inducing ligand (TRAIL). Ferroptosis induces the expression of the apoptosis modulator p53 upregulated modulator of apoptosis (PUMA), yet this modulator alone does not initiate apoptosis, but rather exacerbates an already active process [99]. Therefore, a similar chain of events, where an already activated apoptosis is intensified, can potentially explain the role of ferroptosis in our model. We wanted to elucidate the downstream cascade that results in apoptosis and clarify how ferroptosis could play a part in this process. Apoptosis essentially consists of two different pathways: the intrinsic and extrinsic pathways. In the intrinsic, or mitochondrial pathway of apoptosis, effector caspases are activated after a signaling cascade involving mitochondrial outer membrane permeabilization, causing the release of cytochrome c and the activation of caspase-3 that orchestrates the death of the cell [100]. In the extrinsic, or death receptor pathway of apoptosis, ligation of death receptors on the cell surface leads to the formation of a death-inducing signaling complex, which includes FADD and caspase-8 [101]. We showed that the classic intrinsic apoptosis pathway exists in our model since mitochondrial superoxide formation (Figure 2A), mitochondrial membrane permeation (Figure 2B) and caspase-3 activation (Figure 2D) were induced. Concordantly, all these processes were inhibited with the pan-caspase inhibitor ZVAD (Figure 2A–D). Although ZVAD abrogates apoptosis, it exacerbates damage to BLEC functionality [43] indicating that the intrinsic pathway is not directly responsible for the damage to barrier integrity. The extrinsic pathway appears to also be induced since we have recently shown that caspase-8 inhibition reduced cell toxicity [44]. As mentioned earlier, unlike Fer-1, the iron chelator DFO rescued BLEC from both cell death and apoptosis (Figure 3A,C). Even though DFO inhibited superoxide formation in the mitochondria (Figure 3F) it seems to inhibit apoptosis through the extrinsic pathways since it exacerbated mitochondrial membrane permeation (Figure 3D), an essential step in the intrinsic pathway. Even though we had elucidated the apoptotic pathways and their kinetics, the process responsible for damage to barrier functionality still eluded us. We turned our attention to possible changes in iron since ferroptosis has been reported to deregulate iron homeostasis through manipulation of iron regulatory proteins such as ferritin, transferrin, and transferrin receptor [102,103,104]. Additionally, ferroptosis-induced degradation of ferritin resulted in an accumulation of the cellular labile iron pool [102]. Indeed, a rapid and dramatic increase in the labile iron pool was detected upon insult, while DFO slightly yet significantly reduced this upsurge (Figure 3B). Iron overload is associated with loss of BBB integrity in an in-vivo model of transient forebrain ischemia in rats [105] and has been reported to induce endothelial cell toxicity in an in-vitro model of secondary brain injury after brain hemorrhage [74]. We found that DFO not only rescued BLEC viability (Figure 3A) but also reduced permeability and restored TEER (Figure 3G). Furthermore, the addition of FAS to directly increase the labile iron pool resulted in damage to BBB integrity, yet did not significantly affect BLEC cell viability (Figure 4D), demonstrating that a deregulation in the endothelial iron homeostasis might be crucial for BBB functionality with no effect on cell viability. Iron homeostasis is a tightly regulated process, and the iron sensing proteins iron regulatory proteins 1 and 2 (IRP1 and IRP2) regulate the expression of proteins involved in iron uptake, release and storage. IRPs regulate ferritin H and ferritin L translation and thus iron storage, as well as transferrin receptor 1 (TfR1) mRNA stability, thereby regulating the influx of iron into the cell [106]. Further studies are needed to gain a deeper understanding of what mechanism is responsible for the increase in the iron labile pool by evaluating the expression of proteins such as ferrtin, transferrin receptor, and ferroportin. To elucidate the mechanism by which DFO rescues BBB integrity, we analyzed the expression of the adherens junction protein Ve-cadherin. DFO abrogated Ve-cadherin decrease in protein expression (Figure 4A) but not mRNA expression (Figure 4E), indicating a post transcriptional Ve-cadherin rescue. To find a link between DFO and Ve-cadherin, we investigated the iron regulated HIF2α. HIF2α has been reported to regulate Ve-cadherin expression directly by targeting its promoter in cardiac endothelial cells [73] or indirectly by targeting the promoter of VE-PTP in lung microvascular endothelial cells. Ve-PTP stabilizes Ve-cadherin by preventing its internalization and degradation [72]. Yet, the role of HIF2α in brain endothelial cells and BBB permeability has not been discovered. We found here that DFO significantly increased HIF2α expression 24 h post insult (Figure 4B). To establish the regulatory role of HIF2α on BBB integrity we applied a specific HIF2α inhibitor, which exclusively targets HIF2α but not HIF1α [51]. This inhibitor abrogated both DFO permeability and TEER rescue while having no significant effect on either DFO inhibition of cell toxicity (Figure 4C) or mitochondrial superoxide formation (Figure 4H), strengthening our hypothesis that BLEC viability and integrity are independently regulated by two separate pathways. To validate the role of the iron liable pool in damage to barrier functionality through HIF2α expression, we replaced PX with the classic labile iron pool inducer ferrous ammonium sulfate [74]. While endothelium toxicity was not significantly affected, damage to BBB functionality was evident by enhanced permeability and reduced TEER. This damage was reversed by DFO, while the HIF2α inhibitor abrogated the effect of DFO (Figure 4D). These results reiterated our theory that the barrier integrity is regulated by iron homeostasis in BLEC through the HIF2α—Ve-cadherin axis (Figure 6A). Surprisingly, the HIF2α inhibitor had no significant effect on Ve-cadherin mRNA or total cell protein expression (Figure 4E,F) but exclusively abrogated DFO rescue of Ve-cadherin expression on the cell surface (Figure 4G). A similar phenomenon has been recently reported with TNFα and its effect on Ve-cadherin cell surface expression in HUVEC [107]. It has long been established that acute hypoxia induces BBB permeability [108,109]. Conversely, moderate chronic hypoxia has been shown to enhance BBB stability through the expressions of laminins [110]. HIF1α as opposed to HIF2α has a negative correlation to BBB integrity [111,112]. This can be explained by their opposing roles; HIF1α governs the acute adaptation to hypoxia, whereas HIF2α activity is induced later [113], and this creates a transitional switch between the expression of the HIF1α acute hypoxia targeted genes to the expression of the HIF2α chronic hypoxia targeted genes. The present discovery of a novel HIF2α—Ve-cadherin brain endothelium axis that regulates BBB integrity through iron homeostasis can shed light on the molecular mechanisms involved in the maintenance of BBB integrity upon hypoxia-independent injuries and might furthermore indicate the pathways that are responsible for enhanced BBB integrity in moderate chronic hypoxia. We speculate that laminin could be the missing link in our axis that enables HIF2α to regulate Ve-cadherin surface expression since it has been reported that chronic mild hypoxia increases expression of laminins 111 and 411 and the laminin receptor α6β1 integrin at the BBB [110]. Furthermore, laminin 511 was shown to induce Ve-cadherin localization to cell-cell borders via β1 and β3 integrins [114]. Further studies are needed to elucidate the role of laminins and their receptors and to gain a deeper understanding of the molecular mechanisms of HIF2α regulated BBB integrity. Iron homeostasis balance is an essential component of cellular physiology and is a delicately balanced process. On the one hand, it is involved in critical intracellular processes, including DNA synthesis and cellular respiration. In contrast, iron overload results in ROS formation and oxidative stress through the Fenton reaction [115,116,117]. We therefore sought to find another compound, which will not act as a classic chelator. We have synthesized and screened more than 30 new compounds (not shown) and identified a nitroxide, JP4-039, which is a mitochondrial targeted antioxidant and free radical scavenger [118,119], as a potential candidate. It has been demonstrated that JP4-039 in combination with a bone marrow transplant, increased tight junction protein expression and barrier integrity in epithelial intestinal cells of mice who had been exposed to total body irradiation without affecting cell viability [120]. JP4-039 had multifaceted beneficial effects in our BBB injury model; it inhibited mitochondrial superoxide formation, rescued BLEC cell death via apoptosis inhibition and most importantly, it protected BBB integrity by attenuating BBB increased permeability and TEER decline (Figure 4). The precise molecular mechanism of JP4-039 still needs to be elucidated; nevertheless, we have identified a novel non-toxic chemical that both protects BLEC viability and integrity, with the potential of incorporating it as a therapeutic compound in treatments of diseases associated with BBB breakdown.

## 5. Conclusions

In conclusion, using an established human BBB model, we have shown that chemical insult rapidly induces lipid peroxidation and a ferroptosis-like event. This event potentially enhances separate pathways that result in apoptosis through both the extrinsic and intrinsic pathways (Figure 6B). More importantly, we have demonstrated that the source of BBB breakdown in our model is not a direct result of ROS related processes but rather an imbalance induced in the cellular iron homeostasis of BBB endothelium. We have furthermore elucidated regulation of BBB integrity through the iron dependent HIF2α—Ve-cadherin pathway and that the FDA approved DFO used to treat acute iron poisoning and hemochromatosis in clinic [121], attenuates damage to BLEC viability and barrier integrity by upregulating this pathway (Figure 6A). These results can help clarify the involvement of iron in BBB breakdown in the early stages of neurodegenerative diseases since it has been reported that BBB dysfunction is an early biomarker of neurodegeneration [7] and that there is a clear correlation between iron overload and neurodegenerative diseases [122]. Moreover, a novel compound, JP4-039, was found to treat BBB breakdown. We expect that these findings can be used to assist in developing a viable treatment to attenuate BBB damage, thereby inhibiting BBB-associated disease progression.

## Data Availability

The datasets used and/or analyzed during the current study are available from the corresponding author on reasonable request.
